# Neutrophil extracellular traps in cancer

**DOI:** 10.1002/mco2.647

**Published:** 2024-07-15

**Authors:** Yuxi Ma, Jielin Wei, Wenshan He, Jinghua Ren

**Affiliations:** ^1^ Cancer Center Union Hospital Tongji Medical College Huazhong University of Science and Technology Wuhan China; ^2^ Institute of Radiation Oncology Union Hospital Tongji Medical College Huazhong University of Science and Technology Wuhan China; ^3^ Hubei Key Laboratory of Precision Radiation Oncology Wuhan China; ^4^ Department of Breast and Thyroid Surgery Union Hospital Tongji Medical College Huazhong University of Science and Technology Wuhan China

**Keywords:** cancer, NETosis, NETs formation, neutrophil, neutrophil extracellular traps

## Abstract

Neutrophil extracellular traps (NETs), which consist of chromatin DNA studded with granule proteins, are released by neutrophils in response to both infectious and sterile inflammation. Beyond the canonical role in defense against pathogens, the extrusion of NETs also contributes to the initiation, metastasis, and therapeutic response of malignant diseases. Recently, NETs have been implicated in the development and therapeutic responses of various types of tumors. Although extensive work regarding inflammation in tumors has been reported, a comprehensive summary of how these web‐like extracellular structures initiate and propagate tumor progression under the specific microenvironment is lacking. In this review, we demonstrate the initiators and related signaling pathways that trigger NETs formation in cancers. Additionally, this review will outline the current molecular mechanisms and regulatory networks of NETs during dormant cancer cells awakening, circulating tumor cells (CTCs) extravasation, and metastatic recurrence of cancer. This is followed by a perspective on the current and potential clinical potential of NETs as therapeutic targets in the treatment of both local and metastatic disease, including the improvement of the efficacy of existing therapies.

## Graphical abstract

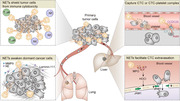

## 1 INTRODUCTION

Neutrophils, the most abundant cell type in circulation, have long been recognized as a critical component of the innate immune response to invading microbial pathogens. As the first responders, neutrophils are rapidly recruited to the sites of injury and infection where they engulf and kill the invading pathogens via phagocytosis, followed by the release of reactive oxygen species (ROS) and granule proteins.^1^ In 2004, a novel antimicrobial mechanism of neutrophils to trap and kill microbes termed NETosis was first identified by Brinkmann et al.^2^ Various pathogens, such as bacteria, fungi, viruses, and parasites can trigger NETosis.^2^, ^3^, ^4^, ^5^ In addition, lipopolysaccharide (LPS), phorbol myristate acetate (PMA), antibodies, immune complexes, cytokines and chemokines, microcrystals, and other physiological stimuli can also induce NETosis.^6^, ^7^, ^8^, ^9^, ^10^ Upon stimulation with pathogens, neutrophils are capable of extruding chromatin and granule proteins to form neutrophil extracellular traps (NETs).^11^ NETs are web‐like structures consisting of highly decondensed chromatin fibers with histones and granule proteins, such as matrix metalloproteinase 9 (MMP9), neutrophil elastase (NE), myeloperoxidase (MPO), cathepsin G (CG), and other antimicrobial proteins.^12^ According to the fate of neutrophils, two different forms of NETosis have been described: lytic NETosis (classical or suicidal NETosis), a special form of programmed cell death distinct from apoptosis and necrosis; and vital NETosis that retains the viability and functions of neutrophils.

The unique structure of NETs makes them fight a wide range of pathogens (bacteria, fungi, viruses, and parasites), all of which could be captured in their tracks and cleared by the antimicrobial proteins and enzymes of NETs. Apart from their involvement in antimicrobials, NETs also contribute to inflammation‐associated carcinogenesis, progression of malignancies, cancer‐related thrombosis, and poor clinical outcomes in different cancer contexts. Following the first description of NETs as a protumorigenic mechanism within Ewing sarcoma in 2013,^13^ there is now a growing consensus from many studies indicating the NETosis processes are responsible for tumor relapse and metastasis in the last few years.

Here, we illustrate the potential triggers and associated signaling pathways that induce the formation of NETs, with a particular emphasis in the context of tumors. Subsequently, mounting evidence highlights that NETs serve as pivotal cues in tumor progression, dormant cancer cells awakening, circulating tumor cells (CTCs) extravasation, and metastatic recurrence. Additionally, NETs are closely involved in the inflammation‐induced disturbance that leads to the development of an immunosuppressive microenvironment, which promotes immune evasion and tumor survival and growth. Considering the functions of NETs mentioned above, NETs may serve as potential prognostic biomarkers, as well as therapeutic targets for cancer.

## 2 MOLECULAR MECHANISMS OF NETs FORMATION

**FIGURE 1:**
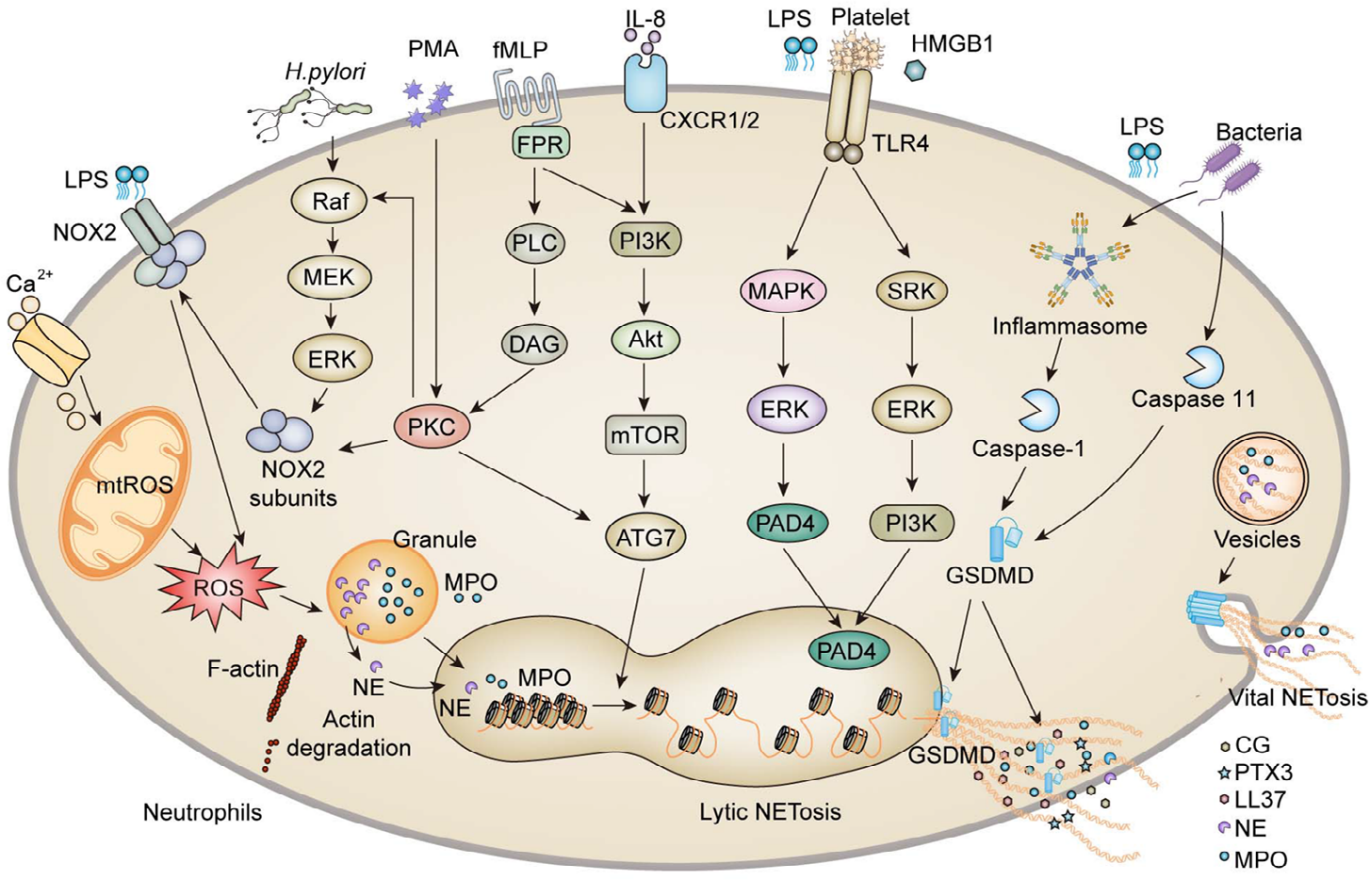
Signaling mechanism of NETosis. Diverse stimuli trigger various molecular signaling of NETosis, including two primary modes, lytic and vital NETosis. Lytic NETosis primarily relies on the NOX2‐dependent ROS generation and independence of ROS. Briefly, receptor activation leads to increased cytoplasmic calcium from both store‐released calcium from the endoplasmic reticulum and the entry of extracellular Ca^2+^. Calcium ions at elevated levels result in the generation of mtROS, increase the activity of PKC and favor the formation of ROS. Directly or indirectly via the Raf–MEK–ERK pathway, PMA can stimulate ROS generation with the participation of PKC, which in turn promotes functional assembly of the cytosolic and membrane‐bound subunits of NOX2. As well, NOX2 can be activated by fMLP via the PLC–DAG pathway to initiate PKC. The generated ROS enables NE and MPO to translocate from granules to the nucleus, where they contribute to chromatin decondensation. Independent of ROS, other receptors, such as TLRs, are also implicated in NETosis. TLR4 is activated by LPS, HMGB1, and platelet components, followed by MAPK–ERK or SRK–ERK–PI3K signaling, which subsequently activates PAD4 to modify histones and decondense the chromatin, allowing it to be ejected. Moreover, cleavage and activation of GSDMD by caspase‐1 and caspase‐11 can also lead to the release of NETs. Another alternative rapid NETs formation, vital NETosis, entails the vesicular transport of DNA from the nucleus to the extracellular environment, along with the release of NE and MPO. ATG7, autophagy‐related protein 7; Ca_2_^+^, calcium ion; CG, cathepsin G; GSDMD, gasdermin D; CXCR 1/2, CXC chemokine receptor 1/2; DAG, diacyl glycerol; ERK, extracellular signal‐regulated kinase; fMLP, formyl‐methionyl‐leucyl‐phenylalanine; FPR, formyl peptide receptor; HMGB1, high‐mobility group box 1; IL‐8, interleukin 8; LL37, 37 amino acid cationic peptide; LPS, lipopolysaccharide; MAPK, mitogen‐activated protein kinase; MEK, MAPK/ERK kinase; MMP‐9, matrix metalloproteinase‐9; MPO, myeloperoxidase; mTOR, mammalian target of rapamycin; mtROS, mitochondrial reactive oxygen species; NE, neutrophil elastase; NOX2, nicotinamide adenine dinucleotide phosphate (NADPH) oxidase 2;PAD4, peptidyl arginine deiminase 4; PI3K, phosphatidylinositol‐3‐kinase; PKC, protein kinase C; PLC, phospholipase C; PMA, phorbol 12‐myristate 13‐acetate; PTX3, pentraxin 3; ROS, reactive oxygen species; SRK, S locus receptor kinase; TLR, Toll‐like receptor.

The release of granules proteins from azurophil granules into the cytosol is a crucial step in the process of NETosis. Azurophil granules contain the protein complex “azurosome,” which includes eight types of proteins. Three of them are highly homologous serine proteases: MPO, NE, and CG.^14^ It has been demonstrated that increased generation of ROS triggers the dissociation of azurosomes, followed by the release of NE and MPO from their granules into the cytosol. As a histone hydrolase, NE degrades cytoskeletal components, which are also present in the nuclear interior, and degrades the lamin and histones, consequently priming the decondensation of chromatin and the disintegration of the nuclear envelope. MPO is an enzyme that promotes histone carbamylation, engaging in azurosome dissociation and enabling protease to be released from granules. Although ROS is highly intertwined with the reaction cascades entailing the release of NETs, there are several exceptions such as chronic inflammation in multiple organ systems, in which the process of chromatin decondensation is ROS unrelated (Figure 1).

### 2.1 Mechanisms governing NETosis

#### 2.1.1 ROS‐related NETosis

The two primary sources of ROS in neutrophils are nicotinamide adenine dinucleotide phosphate (NADPH) oxidase and mitochondria. Rapid generation of ROS (within 20 min) is elicited in neutrophils in response to stimulation with PMA, deoxypodophyllotoxin, (*Staphylococcus aureus (S. aureus)*, or *Beta‐hemolytic streptococci (B. streptococci)*, which can be counteracted by antioxidants.^15^, ^16^ NADPH oxidase (NOX), the major source of ROS, transfers electrons across biological membranes to produce oxidants that support optimal antimicrobial activity.^17^, ^18^ This is a multicomponent enzyme system that actives followed by the assembly of four cytosolic proteins (p47phox, p67phox, p40phox, and Rac2) with the transmembrane proteins (p22phox and gp91phox).^19^, ^20^

In the context of NOX‐dependent NETs generation, the upstream pathways how pathogens trigger NOX2 to release ROS remain poorly defined. Several ROS‐inducible receptors (Box 1) and kinases, such as extracellular‐signal‐regulated kinase (ERK), Mitogen‐activated protein kinase (MAPK)/ERK kinase (MEK), interleukin‐1 (IL‐1) receptor‐associated kinase, protein kinase C (PKC), phosphoinositide 3‐kinase (PI3K) and protein kinase B (PKB, also known as Akt), have been implicated in the biological process.^21^, ^22^, ^23^, ^24^, ^25^, ^26^ Mechanistically, phosphorylation of the NOX subunit p47phox by PKC provokes a conformational rearrangement that exposes the functional domains of NOX and allows the assembly on the membrane.^27^ Hakkim et al.^23^ confirmed that the Raf–MEK–ERK kinase cascade pathway is involved in PMA‐triggered NETosis through activation of NOX. PMA can penetrate the plasma membrane and directly activate PKCα and PKCβ.^28^ According to El‐Benna's model, NOX is invoked when the bacterial peptide N‐formylmethionyl‐leucyl‐phenylalanine attaches to the plasma membrane receptor formyl‐peptide, triggering the protein tyrosine kinases (PTKs)–PI3K signaling pathway, which in turn activates the Raf–MEK–ERK kinase cascade.^29^ Afterward, ERK phosphorylates the cytosolic components (p47phox, p67phox, p40phox), translocating them to the membrane and priming the activation of NOX, in concert with the activated PKC.^29^

In contrast, a different study indicated that ERK was upstream of MAPK signaling during PMA‐induced NETosis and was implicated following ROS generation.^30^ Moreover, the activation of Akt, which is essential for NETosis, depends on NOX‐mediated ROS production.^26^ In addition, the spleen‐associated tyrosine kinase (SYK)–PI3K pathway also mediates ROS production and NETosis.^31^, ^32^ Other triggers, including calcium ionophores, nigericin, specific microorganisms, ultraviolet light, and some crystals, induce NETs formation via the production of mitochondrial ROS rather than NOX.^33^ Therefore, it appears that the signaling routing of NOX‐dependent ROS and mtROS in NETosis is stimulus dependent, and the underlying mechanism has yet to be fully elucidated.

#### 2.1.2 ROS‐irrelated NETosis

The peptidyl arginine deiminase (PAD4) is a neutrophil‐enriched enzyme that catalyzes arginine residues into citrulline on histone 3 in a calcium‐dependent manner for the formation of NETs.^34^ Calcium influx, ROS generation, and intact microtubules are typically required for priming the activation of PAD4.^35^, ^36^ Subsequently, activated PAD4 penetrates the nucleus, where it citrullinates histones to facilitate chromatin decondensation.^34^ This, in turn, diminishes the positive charges in histones and seeks weaken their electrostatic interactions with negatively charged DNA.^37^, ^38^, ^39^ Cl‐amidine, a PAD4 inhibitor, hinders chromatin decondensation and NETosis triggered by Ca^2+^ ionophores or *Shigella flexneri*. Conceivably, neutrophils derived from PAD4‐deficient mice can not generate NETs in response to PMA.^35^

To date, several downstream signaling cascades involved in PAD4‐dependent NETosis have been identified. As such, different PKC isotypes, particularly PKCα, PKCβ1, and PKCζ are integrally involved in PMA‐, ionomycin‐, IL8‐, platelet‐activating factor‐, and Group B *streptococci*‐triggered NETosis.^40^ The cyclin‐dependent kinase 6 (CDK6) and Raf–MEK–ERK pathways are also essential for PMA‐induced NETosis, whereas the SYK–PI3K–mTorc2 pathway facilitates NETosis triggered by monosodium urate crystal and *S. aureus*.^41^ Moreover, Janus kinase 2, a protein kinase that transduces cytokine‐mediated signaling and regulates cellular proliferation, is also implicated in NETosis.^42^ Accordingly, the downstream signaling cascades involved in NETosis are determined by the physiological and biochemical properties of stimulators.^23^, ^41^

Toll‐like receptors (TLRs) are critical components of the innate immune system that recognize pathogen‐ and damage‐associated molecular patterns to mount an immune response. LPS, one of the most prototypical pathogen‐associated molecular patterns, activates platelets via TLR4 and induces neutrophil recruitment, platelet–neutrophil interaction, and ultimately the formation of NETs.^43^ Despite the fact that LPS‐activated platelets initiate the formation of NETosis, which is principally dependent on ROS and regulated by interferon regulatory factor 1.^44^ However, it was also discovered that LPS‐activated platelets via TLR2 and TLR4 could trigger NETosis independently of ROS, with the implicated signaling occurring through ERK, PI3K, and Src kinases.^45^

Damage‐associated molecular patterns (DAMPs) are endogenous molecules released by damaged, dead, or dying cells. High‐mobility group box 1 (HMGB1) is a DAMP that can serve as a ligand for TLR2 and TLR4 to induce NETs.^46^ Multiple investigations have shown that HMGB1 is highly prone to binding with receptors for advanced glycation end products (RAGE), TLR2, TLR4, and TLR9.^47^, ^48^, ^49^ Tadie et al.^50^ discovered that HMGB1 contributes to promoting NETs formation through a TLR4‐dependent mechanism both in vitro and in vivo.^51^ Zhou et al.^52^ confirmed consistently in lung cancer that p38 MAPK and ERK, two molecules downstream of TLR4, were activated during NETs formation. Furthermore, it has been reported that HMGB1 induces NETs formation via tumor necrosis factor (TNF) and receptor‐interacting‐protein kinase‐1 kinase activity during skin carcinogenesis.^53^ As well, TLR9 is an essential DAMP sensor that mediates the formation and proinflammatory impact of NETs. TLR4/9 activation and subsequent cyclooxygenase‐2 upregulation were identified by Qin's team as the critical signaling in NETs‐triggered metastasis.^54^

The caspase‐dependent gasdermin D (GSDMD) protein is the pivotal element for assembling pores in the neutrophil membrane, allowing the giant complex of decondensed chromatin tightly attached with granule protein to pass through at the last stage of NETosis.^55^, ^56^ Activation of GSDMD probably leads to the formation of pores not only in the plasma membrane but also in the nuclear membrane.^57^ Several stimuli contribute to the activation of caspase‐1 via the NLR family pyrin domain containing 3 inflammasome pathway.^58^ Upon activation by the inflammasome, caspase‐1 typically cleaves GSDMD to release cytokines, including IL‐1,^58^ and this pathway has recently been demonstrated to drive NETosis.^59^ Unexpectedly, cytosolic LPS and gram‐negative bacteria activate caspase‐4/11‐inflammasome signaling and trigger GSDMD‐dependent NETosis.^55^ Caspase‐11 and GSDMD facilitate nuclear delobulation and DNA expansion via nuclear membrane permeabilization and histone degradation.^55^ Intriguingly, granule protein NE can also activate GSDMD, which in turn generates pores in the granule membrane, boosts NE release into the cytoplasm and NETs formation. These findings identify a positive feed‐forward loop in the GSDMD–NE pathway to accelerate NETosis.^56^

### 2.2 NETs formation in the context of cancer

**FIGURE 2:**
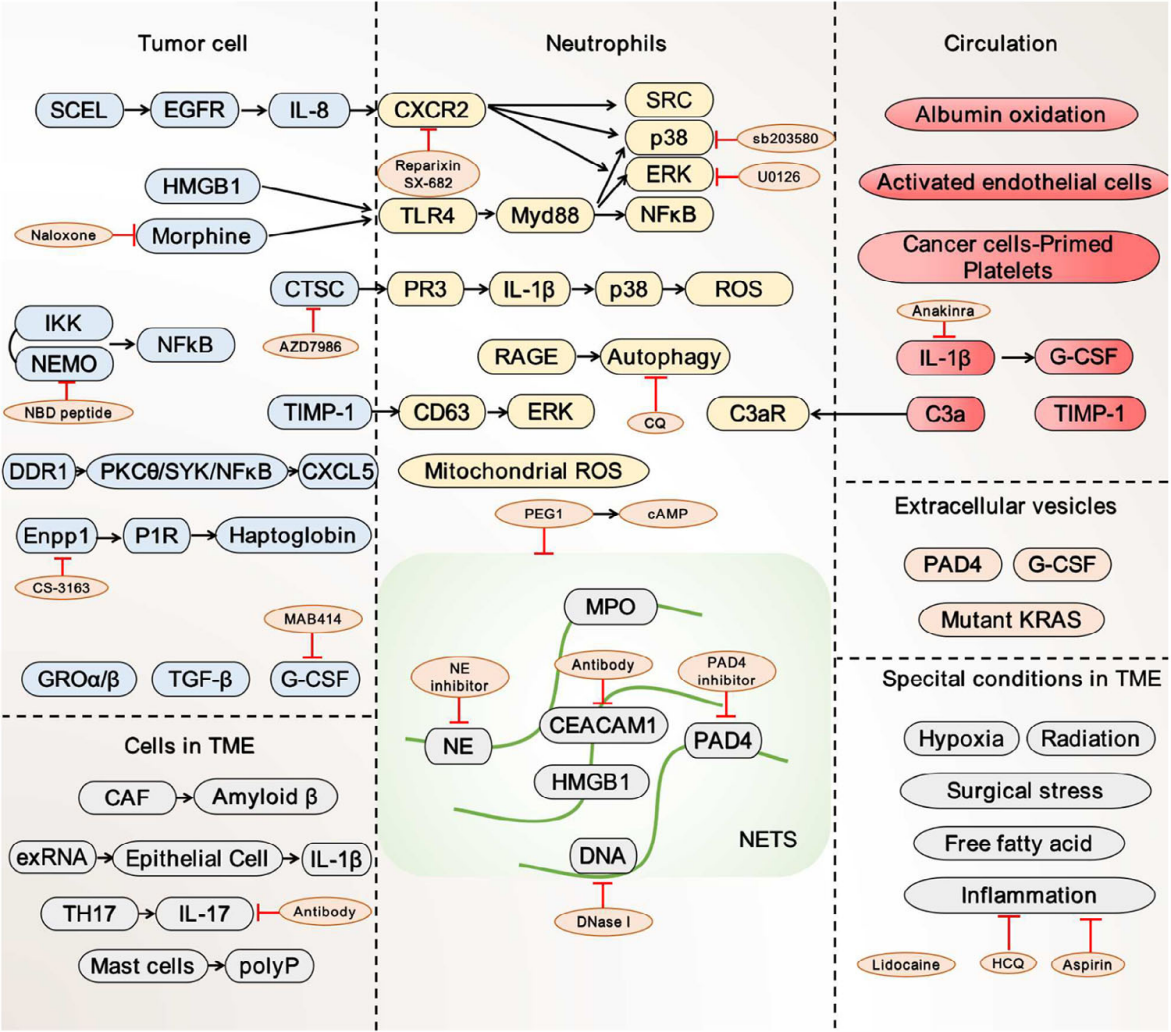
Multiple tumor‐derived factors contribute to NETosis. The formation of NETs is effectively encouraged by cancer cells, surrounding stromal and inflammatory cells in tumor tissues, secreted EVs in TME, as well as soluble mediators in the bloodstream. The tumor‐specific microenvironment including hypoxia, radio‐ and surgical stress, and inflammation acts as a ‘fertile soil’ to accelerate NETs development. These stimulators draw in neutrophils and initiate various signaling pathways in neutrophils that are responsible for NETosis, most of whose factors could be targeted by an expanding number of inhibitors. EVs, extracellular vesicles; TME, tumor microenvironment.

Tumors are often viewed as wounds that do not heal, in which cancer‐related inflammation typically occurs. Cancer cells, as well as surrounding stromal and inflammatory cells, are well‐orchestrated to form an inflammatory tumor microenvironment (TME), ultimately favoring the proliferation and survival of malignant cells. Various soluble factors derived from both non‐neoplastic and neoplastic cells, including cytokines, chemokines, and growth factors have been implicated in NETs formation (Figure 2).

#### 2.2.1 Tumor cell initiates NETs formation

In preclinical models of many invasive cancer cells, including breast(4T1, BT‐549, and D2.A1),^60^, ^61^ pancreatic (AsPC‐1),^62^ and colonic (HT‐29, CT26, and LS174T)^63^ cell lines, NETosis has been indicated to be directly induced in the coculture system in vitro. However, not all cancer cells induce NETs formation. Cell lines such as 4T07, D2.0R, and MCF‐7, for instance, cannot trigger the production of NETs.^60^, ^61^ These mechanisms vary in different tumor types and depend on the soluble factors secreted by tumor cells.

Among the tumor‐derived factors that act on neutrophils, the cancer cell granulocyte‐colony‐stimulating factor (G‐CSF) is considered the primary trigger of NETosis. G‐CSF is a glycoprotein that stimulates the release of neutrophils from the bone marrow, as well as regulates the generation of ROS and subsequent NETosis.^60^, ^64^, ^65^ In head and neck cancer patients, G‐CSF was identified to directly stimulate neutrophils to release NETs.^66^ Similarly, NETs formation has also been shown to be significantly associated with elevated levels of G‐CSF secreted from 4T1 cells, thereby leading to breast cancer metastasis.^64^ NETosis can be elicited by G‐CSF enriched conditioned medium from ovarian cancer cells, thus facilitating omental metastasis by the construction of a favorable environment for distant colonization.^65^, ^67^

IL‐8 (also known as CXCL8) is another crucial activator and chemoattractant for neutrophils and has recently been implicated in NETs induction.^68^ In cell culture assays, cancer cells can induce NETsosis by producing ligands for CXCR1 and CXCR2 (such as CXCL‐1, ‐2, ‐5, ‐6, and ‐8). It was found that NETs density within tumor tissue had a positive correlation proportional to the abundance of IL‐8 protein. Intriguingly, NETs are inversely correlated with CD8^+^ tumor‐infiltrating lymphocytes in non‐small cell lung cancer (NSCLC), bladder cancer, and metastatic melanoma.^69^ Conditioned medium from anaplastic thyroid cancer cells primed neutrophils to release NETs through the secretion of IL‐8.^70^ Similarly, both cancer cells and plasma from advanced‐stage diffuse large B‐cell lymphoma patients carrying high IL‐8 resulted in the substantial formation of NETs.^71^ Blockade of NETs formation through depletion of IL‐8 prevents omental metastasis of ovarian cancer patients.^67^ Apart from neutrophils, IL‐8 attracts myeloid‐derived suppressor cells (MDSCs), a type of protumorigenic immune‐suppressing cells, to reduce T lymphocytes activities.^68^ The biological mechanism of IL‐8‐mediated T‐cell suppressor activity depends on the formation of NETs. Teijeira et al.^72^ described their study on both neutrophils and MDSCs showing that CXCR1 and CXCR2 agonists, particularly IL‐8, induced extrusion of NETs around tumor cells. Besides IL‐8, other glutamic acid–leucine–arginine (ELR)‐positive chemokines that bind to CXCR1/CXCR2 can also promote the recruitment of neutrophils and contribute to NETs formation.^63^

Some other modulators, such as growth‐regulated oncogene‐α and ‐β (GROα and GROβ), HMGB1, transforming growth factor‐β (TGF‐β), tissue inhibitor of metalloproteinases‐1 (TIMP‐1), discoid domain receptor 1 (DDR1), haptoglobin (Hp), and extracellular RNA (exRNA), could be generated by malignancies, resulting in neutrophil activation and NETs formation. GROα and GROβ that are secreted from ovarian cancer cells invigorate NETs formation and promote metastasis.^67^ TGF‐β that originated from oral lichen planus has been implicated in the development of oral cancer by facilitating the generation of NETs.^73^ Meanwhile, a recent study concluded that tumor‐derived protein TIMP‐1‐induced NETs formed by the interaction with its receptor CD63 and subsequently activated ER.^74^ Notably, high expression of DDR1 on pancreatic ductal adenocarcinoma (PDAC) cells has the potential to mediate communication between cancer cells and neutrophils. In this process, CXCL5, which acts as a downstream effector, is required for DDR1‐induced neutrophil infiltration and NETs formation.^75^ Recent research on the crosstalk between NETs and exRNA revealed that higher levels of exRNA released by lung tumor cells indirectly promote NETosis by activating bronchial epithelial cells (ECs), which in turn accelerates exRNA release from ECs.^76^ An additional TLR4 ligand, morphine, exacerbated lung cancer cell‐induced NETs, which could potentially be prevented through treatment with the antagonist Naloxone.^52^ Most recently, proinflammatory protein Hp secreted from Enpp1^high^‐tumor cells was demonstrated as a potent chemoattractant for PMN‐MDSC, as well as a novel and unforeseen trigger of NETosis to promote local relapse.^77^

Intriguingly, several components of NETs further encourage neutrophil infiltration and NETs formation. HMGB1 is capable of inducing NETs in addition to acting as a neutrophil chemotactic agent, especially in the stressed TME.^78^ As a component of NETs, HMGB1 is released from neutrophils undergoing NETosis, which has allowed for additional TLR4‐ and TLR9‐dependent pathway activation.^79^ The blockade of CG, a cysteine protease located on NETs, can inhibit NETs extension via decreased release of ROS and IL‐1β.^80^

Besides these soluble factors, extracellular vesicles (EVs) secreted from tumors are broadly investigated as possible triggers for NETosis. EVs are membrane‐encapsulated vesicles carrying bioactive factors that are responsible for the communication between tumor cells and surrounding cells.^81^, ^82^ Here, the authors described thus far unknown roles of pro‐NETotic factors enriched EVs in NETosis by transfer of cargoes from donor to recipient neutrophil. The direct stimulatory impact was reported by Leal et al.,^83^ in which 4T1‐derived EVs activate neutrophils from G‐CSF treated mice to form NETs. In vivo intravenous injection of these tumor‐derived EVs dramatically increased cancer‐associated thrombosis, a common complication in patients with cancer linked to excessive NETs formation. EVs containing KRAS, a driver gene that is frequently altered in colorectal cancer, contribute to the formation of NETs.^84^ To explore the mechanism of mutant KRAS in NETs formation, Dong et al.^85^ showed that KRAS proteins loaded within exosomes were uptaken by neutrophils to produce IL‐8 and NETs. Thus, the potential and capabilities of EVs to stimulate NETs formation may vary depending on their cargoes.

#### 2.2.2 Tumor microenvironmental regulation of NETosis

TME is made up of nonmalignant cells, including immune cells, cancer‐associated fibroblasts (CAFs), and pericytes; as well as noncellular components such as cytokines and the extracellular matrix (ECM). Figure 2 shows several putative pro‐NETotic factors associated with cancer progression in TME. CAFs, the most prevalent stromal populations in TME executing multiple protumor functions, have recently been discovered as a major mediator in lytic NETosis. Munir et al.^86^ discovered that Amyloid β, a protein linked with neurodegenerative illness, was the crucial CAF‐derived component responsible for the induction of NETosis in situ through the CD11b receptor on neutrophils. Systemically, Amyloid β drove NETs into the bloodstream and bone marrow to support metastatic colonization. NETs play a role in the recruitment and activation of CAFs to initiate micrometastasis formation of PDAC and vice versa.^87^ Additionally, studies also explored the interplay between ECs and NETs in TME and discovered that activated bronchial ECs evoked NETs formation. It makes it easier to pinpoint how ECs function in cancer progression in TME.^76^ Besides, immune suppressor IL‐17 produced by TH17 cells contributes to resistance to immune checkpoint inhibitors (ICIs) by generating neutrophil infiltration and NETs formation in pancreatic tumors. The mechanism was investigated in mice lacking PAD4 to find a decline in NETs, increased recruitment of CD8^+^ T cells, and lower tumor size when administrated with a programmed cell death protein 1 (PD‐1) blocker.^88^ Moreover, it is desired for CD68^+^ mast cells to engage with neutrophils, resulting in NETs formation through inorganic polyphosphate, an abundantly present molecule in the colorectal cancer TME.^89^ Taken together, these findings highlight the potential of stromal cells to promote NETosis.

TME remains a complex and dynamic process under different conditions. The rapid growth of the tumor and abnormal tumor vasculature generate a hypoxic environment, which could promote the development of NETs. In the hypoxic liver tumor region, Tohme et al.^79^, ^90^ observed a higher rate of tumor‐infiltrating neutrophils and NETosis compared with normal background liver. In vitro, condition medium from hypoxic MC38 cells provoked the release of NETs.^79^, ^90^ Notably, after exposure to either cigarette smoke or nasal instillation of LPS, the induced inflammatory environment of tumor potential disseminated sites caused NETs formation and accelerated dormant cancer cells to form lung metastases in mice.^61^ In the context of solid tumors, localized control by surgery continues to be a crucial curative option. Postoperative complications especially systemic inflammation remain essential contributors to tumor recurrence. Studies revealed that severe infections triggered NETs following cecal ligation and puncture^90^, ^91^ or ischemia and reperfusion injury.^79^ To investigate the influence of postoperative abdominal infectious complications on NETs formation, Zhao et al.^92^ developed a modified infection model called cecal puncture without ligation and discovered that AIC activated neutrophils to release NETs, allowing gastric cancer cells to form live metastasis.

As significant contributors to tumor biology, tumor‐associated neutrophils (TANs) have been widely described.^93^ In accordance with cytokine production patterns and effector functions, TANs are able to polarize into two populations: the antitumorigenic “N1” phenotype or the protumorigenic “N2” phenotype.^94^, ^95^ In case of sterile inflammation, N1 neutrophils predominate during an acute phase and provoke an exaggerated inflammatory reaction through the secretion of NETs. However, as later phases, there is a progressive increase in the proportion of N2 neutrophils, which exerts a beneficial influence by means of the secretion of anti‐inflammatory factors.^96^ N2 neutrophils are prevalent in types of cancer. An N2‐like gene expression profile was identified through sequencing analysis of CTC‐associated neutrophils, suggesting that not all neutrophils within the clusters are involved in the formation of NETs.^94^ In addition, NETs are more likely to develop in a population of aged neutrophils (CXCR4^+^CD62^Low^).^97^ In the lung premetastatic niche of breast cancer, this subset of neutrophils gathered and ensnared tumor cells by producing vital NETs. Actually, there is a lack of concrete evidence regarding potential distinctions in NETs formation between different types of neutrophils in cancer.

#### 2.2.3 The role of blood components in NETs generation

Neutrophils are also modulated by components existing in circulation. Albumin is the most abundant endogenous antioxidant in the plasma, and as a result, in charge of regulating the redox state of the plasma. ROS within circulating neutrophils could be scavenged by albumin, which is significantly reduced in cancer patients. As a consequence, neutrophils can form NETosis by accumulating ROS due to albumin oxidation in the plasma, which is associated with pulmonary metastasis in patients with head and neck cancer.^98^ Vascular ECs construct the walls of the tumor vasculature, enabling nutrition supply as well as chemotherapeutic administration.^99^ To enable neutrophils recruitment and adherence to the specific region, activated ECs express various adhesion molecules on their surface including P‐selection, E‐selection, and ICAM‐1, and produce cytokines such as IL‐8 to facilitate NETs formation.^100^, ^101^ Meanwhile, it appears that NETs cocultured with ECs over a long period results in ECs damage.^101^ Thus, the administration of either PAD4 inhibitors or DNase I subverts NETosis without increasing the risk of blood vessel damage and atherosclerosis.^102^, ^103^ Prior to initiating NETs formation, platelet activation has been shown in circulation upon encountering tumor cells. Platelets primed by pancreatic tumor cells can also attract neutrophils and stimulate NETosis, which occurs in a vital process. Upon activation, the adhesion molecule P‐selection on the surface of platelets binds to the neutrophil receptor P‐selection glycoprotein ligand‐1 (PSGL‐1) to foster NETosis. High expression of HMGB1 on the platelets also plays a crucial role in NETs release through interaction with neutrophil TLR4 or RAGE. NETs formation could be prevented by treatment with the inhibitors of P‐selection, PSGL‐1, or RAGE. Complement C3a has also been identified as a mediator for platelet aggregation, subsequently resulting in thrombosis and NETosis through binding to C3aR.^104^ In circulation, IL‐1β promotes a prothrombotic state in 4T1 tumor‐bearing mice through G‐CSF, while its antagonist anakinra decreases NETs markers such as MPO and circulating cell‐free DNA.^105^ Reciprocally, NETs are also capable of trapping and activating platelets, hence resulting in thrombus formation conducted by a whole blood perfusion assay.

## 3 THE ROLE OF NETs IN TUMORIGENESIS AND TUMOR PROGRESSION

### 3.1 NETs promote inflammation‐associated carcinogenesis

Chronic inflammation predisposes tissue to the development of cancer.^106^ In addition to the immune surveillance function in the early stages of tumorigenesis, inflammation is also considered one of the hallmarks of cancer that can provide biologically active molecules and contribute to the development of other hallmarks of cancer, such as genetic instability and angiogenesis.^107^ Of note, obesity, a state of chronic low‐grade systemic inflammation, has been linked to an increased risk of more than a dozen types of cancer, as well as a poorer prognosis and shorter life expectancy. Along with metabolic modifications, obesity is characterized by the infiltration of leukocytes into adipose tissue and increased secretion of proinflammatory cytokines. Obesity‐associated nonalcoholic steatohepatitis (NASH) is currently the most important leading cause of hepatocellular carcinoma (HCC) among the noncirrhotic risk factors in developed countries.^108^, ^109^ The current view is that macrophages, including liver‐resident macrophages (Kupffer cells) and infiltrating macrophages derived from monocytes, mediate the activation of innate immune supporting inflammation, as well as the development and progression of NASH, fibrosis, and HCC. Efferocytosis is a crucial process by which neutrophils engulf apoptotic tumor cells, undergo respiratory explosion, and generate NETs. Neutrophils that are engaged in efferocytosis release a range of soluble substances, including cytokines and chemokines, which also contribute to inflammation‐associated carcinogenesis.^110^ However, the drivers of procarcinogenic inflammation linked to tumorigenesis remain largely unresolved.

A mechanistic link between NETs and the evolution of NASH and its progression to HCC has recently emerged.^111^ Data from a hospital‐based study involving 86 patients in the United States showed elevated NETs marker MPO–DNA in their preoperative serum samples of patients with NASH. Along with the findings, they found hepatic neutrophil infiltration and NETs formation occurred early in the onset of NASH in a high‐fat diet and streptozotocin (STAM) mouse model. Elevated free fatty acids in NASH, especially palmitic (C16:0) and linoleic (C18:2), are the main stimuli contributing to NETs formation. In response to neutrophil activation and NETs formation, blood‐derived monocytes are recruited to the livers and differentiate into proinflammatory macrophages. When infiltrating macrophages became numerous, the increased production of proinflammatory cytokines such as IL‐6 and TNF‐α was found, leading to the amplification of inflammation. Consistently, blocking NETs by inhibitor DNase I or using PAD4^−/−^ mice ameliorated the inflammatory environment with persistently reduced IL‐6 levels and the development of HCC, indicating that the contribution of NETs to the initiation of inflammation favors the development of HCC. Although the types of molecules and the underlying mechanisms by which NETs attract macrophages and polarize them towards a proinflammatory phenotype remain unknown, this study exemplifies that aberrant NETs contribute to the generation of monocyte‐derived inflammatory macrophages, which together lead to the onset of NASH and development of HCC.

**FIGURE 3:**
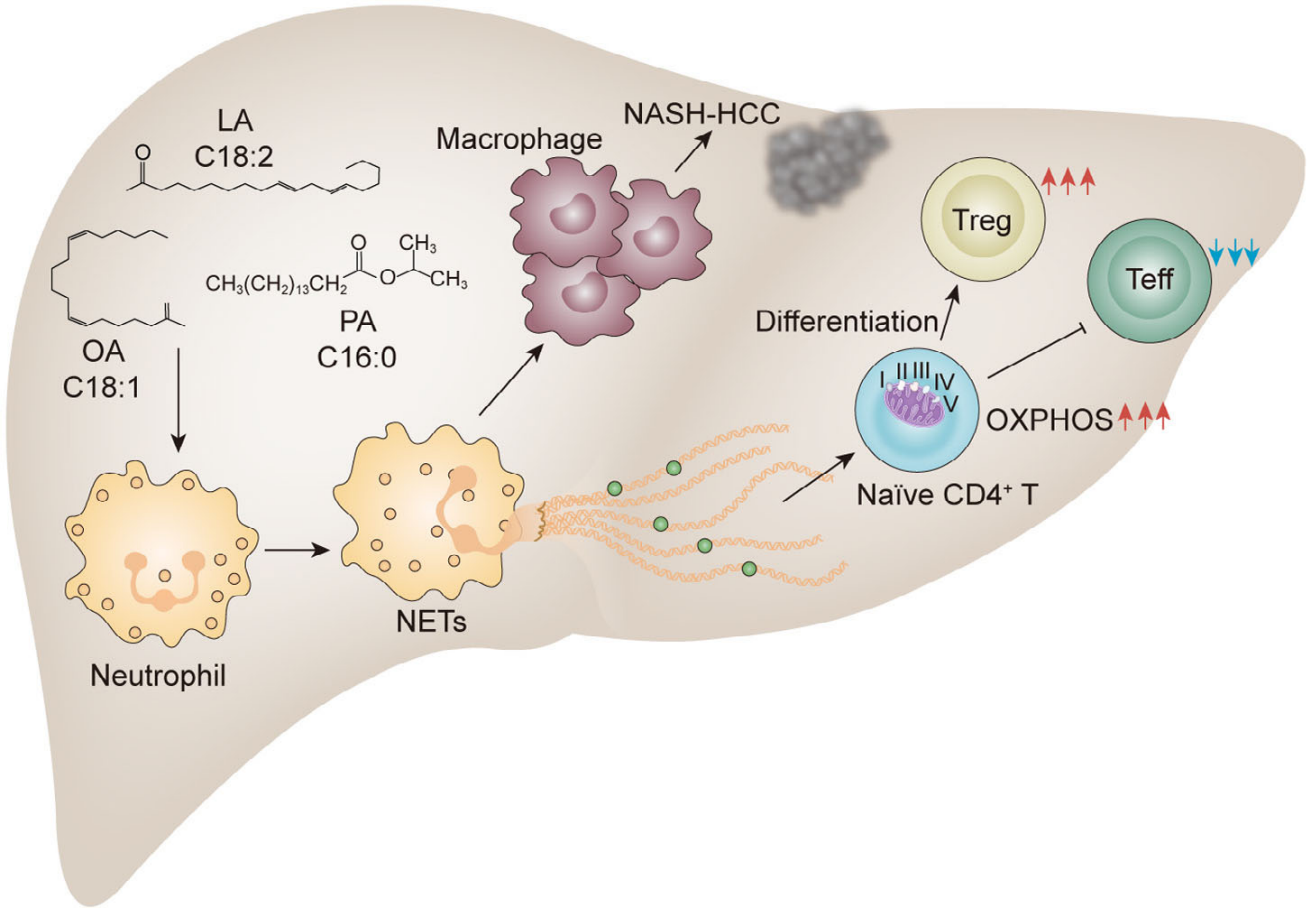
NETs exacerbate the evolution of nonalcoholic steatohepatitis and its progression to hepatocellular carcinoma. NASH is characterized by hepatic steatosis, cellular inflammation, and hepatocyte damage with or without fibrosis/cirrhosis. Hepatic chronic inflammation, mediated by both innate and adaptive immune cells, is the key factor involved in the progress of NASH and NASH–HCC. Recent findings on NASH–HCC pathogenesis have expanded our understanding of its complexity including the contributory role of infiltrated neutrophils. Elevated free fatty acids present in a murine model of NASH–HCC, including oleic, palmitic and linoleic acids, activate NETs formation, leading to the recruitment of infiltrating macrophages, which amplify hepatic inflammation and progression to HCC by the release of proinflammatory cytokines. The other dominant theory proposes that NASH‐induced NET interacts with naïve CD4^+^ T cells within the inflammatory microenvironment, dictating the substantial differentiation towards Treg to foster immune suppression and NASH–HCC progression. To guide the differentiation, the functional protein compositions foster the expression of mitochondrial oxidative phosphorylation‐related genes in naïve CD4^+^ T cells, resulting in metabolic reprogramming that is required for both Treg maintenance and effector T cells attenuation. Pharmacological and genetic blockade of NETs formation in a murine model of NASH–HCC reverses macrophage infiltration, Treg differentiation and subsequent tumor progression. NETs, thus, are critical to the inflammation‐driven tumor promoting environment during NASH–HCC development. HCC, hepatocellular carcinoma; LA, linoleic acid; NASH, nonalcoholic steatohepatitis; NETs, neutrophil extracellular traps; OA, oleic acid; PA, palmitic acid; Teff, effector T cells; Treg, regulatory T cells.

Immune escape is a critical hallmark in solid tumors. Chronic inflammation is also implicated in the perturbation of tissue homeostasis by shaping the liver immune environment, resulting in the escape of malignancies from immune surveillance. A decline in the intrahepatic CD4^+^ T population has been identified as associated with the development of NASH–HCC in both the mouse model and human samples. The current view is that the accumulation of fatty acids in the liver of NAFLD induces the production of mitochondrial ROS, which promotes the loss of CD4^+^ T cells and impairs immune surveillance.^112^, ^113^ More recently, Wang et al.^114^ demonstrated that a selectively increased intrahepatic CD4^+^FoxP3^+^ regulatory T cells (Tregs) in NASH livers even when the total CD4^+^ T‐cell count decreased in STAM model. Additional data from in vitro study indicate that NETs interact with CD4^+^CD44^lo^CD62L^hi^ naïve T cells and reprogram their metabolic process by upregulating mitochondrial oxidative phosphorylation‐related genes, thereby contributing to the differentiation of Tregs. In addition to fostering the differentiation of Tregs, NETs have also been suggested to be capable of impairing the proliferation of effector T cells, resulting in the dysfunctional immune response to hepatocarcinogenesis in the NASH liver microenvironment. Indeed, the spectrum of CD4^+^ T cells, in particular, a dramatic increase in the proportion of Tregs was also found in the other diet‐ and chemical‐induced NASH–HCC mouse model feeding with a high‐fat diet plus diethylnitrosamine during the premalignant stage in NASH livers, rather than the blood or spleen. Likewise, there is a positive correlation between NETs and Tregs in the liver sections of patients with NASH, even after the development of HCC. Altogether, these data delineate that NETs break CD4^+^ T‐cell homeostasis by metabolic reprogramming and tilt the balance toward Tregs, thereby facilitating the dysregulated immune surveillance in the pathogenesis of NASH–HCC (Figure 3).

### 3.2 NETs in tumor progression

**FIGURE 4:**
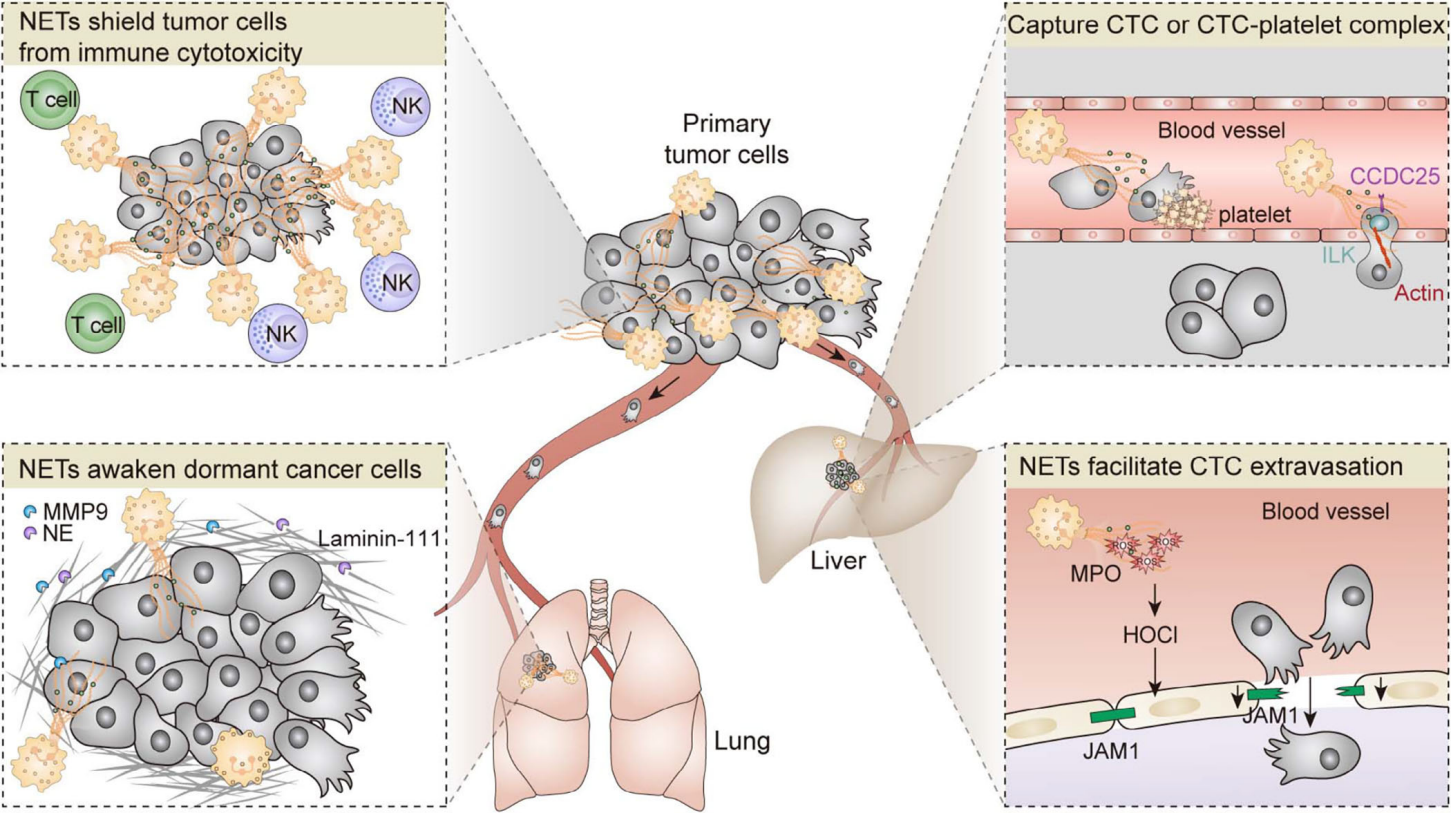
The roles of NETs in therapeutic resistance and metastasis. To support primary tumor growth, the formation of NETs triggered by tumor‐derived CXCL chemokines (CXCL‐1, ‐2, ‐5, ‐6, and ‐8) shield around tumor cells from T cell‐ and NK‐mediated immune attacks. It is possible that these web‐like extracellular structures contribute to the robustness of the tumor, leading to the emergence of resistance to immune checkpoint inhibitors. Once in circulation, NETs capture both CTC and CTC/platelet aggregations directly or indirectly by the CCDC25 receptor–ligand interaction, thus facilitating CTC colonization in distant organs. Along with MPO‐HOCl, ROS that produced during NETosis impairs lung endothelial barrier integrity and facilitates tumor extravasation by reprogramming the expression of the tight junction protein JAM 1 in the endothelium. More intriguingly, MMP9 and NE located on the NETs can awaken dormant cancer cells by degradation of laminin‐111 within ECM. CTC, circulating tumor cell; ECM, extracellular matrix; JAM1, junctional adhesion molecule‐1; MMP9, matrix metalloproteinase 9; MPO‐HOCl, MPO‐derived oxidant hypochlorous acid; NE, neutrophil elastase.

Metastasis is the leading cause of cancer‐related deaths, and there is growing evidence that host immune cells play a critical role in driving tumor progression and distant metastasis. TANs are now recognized not only as simple stromal remodeling cells involved in cancer‐associated inflammation but also as a multifaceted modulator of the immunologic microenvironment,^115^ tumor extravasation,^116^ epithelial‐to‐mesenchymal transition, angiogenesis,^117^, ^118^ as well as macrometastasis formation.^119^, ^120^ In addition to the mechanisms proposed thus far, novel aspects of neutrophils may contribute to cancer progression and metastasis. Accumulating evidence suggests that neutrophils promote colonization and extravasation of disseminated carcinoma cells by their activation and NETs formation (Figure 4).

#### 3.2.1 NETs potentiate metastatic colonization

In the first investigation of the putative role of NETs in cancer progression, the adhesion of cancer cells to neutrophil monolayer pretreated with or without PMA has been investigated in vitro under both static and dynamic conditions.^121^ Enhanced capture of tumor cells by NET‐DNA was observed and this finding was confirmed in the sepsis mouse model, in which NETs deposition in liver sinusoids and pulmonary capillaries during infective systemic inflammation was strongly correlated with the increase of hepatic and pulmonary metastasis 48 h after tumor cells injection.^122^ These findings could potentially explain the clinical phenomenon that severe postsurgical infection is related to adverse oncologic outcomes, which raises the possibility that NETs are a potential therapeutic target.

Besides infective inflammation, sterile inflammation resulting from surgical stress during the perioperative period has also been implied in NETs formation and metastatic progression.^123^, ^124^ Using a murine hepatic ischemia/reperfusion injury model of localized surgical stress, Huang et al.^123^ found that the release of DAMPs during surgical insult promotes NETs formation through TLRs signaling pathway. Subsequently, NETs exacerbate sterile inflammatory liver injury by direct physical damage to hepatocytes or indirect insult via proinflammatory cytokines secreted from Kupffer cells. Since the deposition of NETs in liver sinusoids of mice underwent hepatic ischemia/reperfusion, it is not surprising that surgical stress promotes the adhesion of CTCs and the formation of micrometastatic foci, as described by the same research team thereafter.

As shown by the accumulation of evidence recently, NETs perform crucial roles in tumor metastasis.^125^ A widely acknowledged risk factor, psychological stress significantly influences cancer metastasis, which is in part dependent on NETosis.^126^ In the context of breast cancer lung metastasis, chronic stress stimulates the synthesis of acetylcholine by pulmonary ECs, which subsequently alters the lung premetastatic niche of breast cancer through the promotion of NETosis. Mechanically, chronic stress‐induced neutrophil recruitment into the lung is primarily facilitated by CXCL2, which subsequently promotes NETosis to encapsulate cancer cells in the lung.^127^ What's more, NETs facilitated the migration of colorectal cancer cells without affecting their proliferation with the assistance of NE secreted during NETosis to increase ERK activity. Therefore, a potential therapeutic for tumor metastasis could be the inhibition of NE. The principal component of NETs, NE impacts tumor cell adhesion and subsequently regulates invasion and metastasis of oral squamous cell carcinoma (OSCC). With a comprehensive investigation into its mechanism, pyroptosis, a type of programmed cell death in which NLRP3 is the primary inflammatory complex involved, is inhibited by NETs, thereby instigating OSCC metastasis.^128^, ^129^ Reprogramming of cancer metabolism contributes to multiple stages of the metastatic cascades.^130^ An important metabolic characteristic of metastatic HCC is higher acetyl‐CoA, which increases the expression of the CXCL1 in an H3 acetylation‐dependent manner. As a result, the upregulated CXCL1 increased TANs infiltration and facilitated the formation of NETs.^131^ Several studies reported that multifaceted NET constituents exert an impact on immune cells, thereby indirectly facilitating the tumor metastasis. A study demonstrated that NETs harbored the immunosuppressive ligand PD‐L1, which was accountable for inducing T cell malfunction.^132^ Furthermore, genes associated with exhaustion were upregulated in CD8+ T cells following cocultured with NETs, mediating NSCLC metastasis.^133^ The binding of DNA of NETs to CD8+ T cells via transmembrane and coiled‐coil domains 6 (TMCO6) inhibits antitumor immunity through increasing apoptosis and TGF‐1 secretion, consequently facilitating the progression of HCC.^134^ Furthermore, the investigation of how NETs induce polarization of M2 macrophages has been undertaken.^135^ However, the precise mechanism by which NETs affect immune cells remains inadequately understood, necessitating additional research.

Since an intricate, bidirectional communication between platelets and NETs has been implicated, Ren et al.^121^ charted the capture process with an emphasis on platelets in the context of surgical stress. Although activated platelets were not necessary for NETs formation during hepatic surgery, they facilitated NET‐mediated capture of CTCs and subsequent metastasis by serving as a bridge. Specifically, surgical stress triggered a metastatic cascade in which phosphorylation of platelet ERK5 via the TLR4‐dependent pathways, upregulation of surface integrin GPIIb/IIIa, and subsequent platelet aggregation with CTCs. Such aggregation facilitates NET‐mediated capture of CTCs both in vitro and in vivo. Blocking platelet activation or knocking out TLR4 on platelets protected mice from surgery‐induced metastasis. Thus, this study reveals novel mechanisms by which platelets and NETs coordinate to promote metastasis.

Rather than merely acting as a “trap” for CTCs entrapment, Yang et al.^136^ recently verified that NET‐DNA acts as a chemotactic factor to attract cancer cells via receptor–ligand interactions. By liquid chromatography coupled with mass spectrometry assay, the coiled‐coil domain‐containing protein 25 (CCDC25) on the tumor surface was identified as a NET‐DNA receptor that recognizes the specific molecular structures of extracellular DNA. As a result, ligand‐specific activation of the transmembrane protein CCDC25 triggers the ILK–β‐Parvin–RAC1–CDC42 cascade, which enhances tumor cell motility and eventually promotes cancer metastasis. Importantly, targeting CCDC25 effectively suppresses metastasis of breast cancer in a mouse model. Overall, these findings reveal a mechanism of how NET‐DNA regulates metastatic colonization.

#### 3.2.2 NETs facilitate CTCs extravasation

Extravasation of cancer cells is a critical step in distant metastasis and is thought to be initiated by the disruption of the EC barrier by malignant tumor cells. It is speculated that extravasation of tumor cells involves adhesion to endothelium, regulation of endothelial barrier, and subsequent transendothelial migration (TEM) into the metastatic site.^137^, ^138^ The integrity of the endothelial monolayer is considered critical in metastasis for the dynamic regulation of tumor migration, by permitting or blocking cell movement, thus determining secondary organs targeted by the tumor. In contrast to the well‐established interaction between intravascular tumor cells and endothelium by cell–cell adhesion receptors, blood components in tumor TEM are less clear. When melanoma cells were injected intravenously into mice with elevated blood neutrophils, the tumor cells were retained in the lungs much longer than in control mice.^139^ Similar results were found in another melanoma experimental metastasis model.^139^ The adoptive transfer of normal human neutrophils into mice 1 h after tumor cell injection resulted in the triple retention of melanoma cells in the lungs. It has been reported that neutrophils are recruited to the metastatic site before or shortly after the arrival of tumor cells, where they promote metastasis by facilitating the attachment of tumor cells to ECs and subsequent infiltration into the metastatic site.^140^

A causal relationship between neutrophil and tumoral extravasation in the context of obesity has recently been established, driven by ROS production during NETosis that impairs lung endothelial barrier integrity.^141^ Obesity is a prominent risk factor for breast cancer and a contributor to increased morbidity from metastatic disease. Previous studies have shown that obesity alters the lung myeloid cell landscape, particularly driving neutrophils to traffic to the lungs and promoting breast cancer metastasis.^142^ To explore the relative contributions of obesity‐associated neutrophilia to the extravasation phenotype, McDowell and colleagues^141^ first interrogated the effector cells that manipulate tumor TEM in response to obesity. Pretreatment of ECs, rather than breast cancer cells, with obese serum significantly enhanced TEM compared with lean serum. Enhanced vascular permeability and breast cancer cell extravasation in the lungs were observed in both diet‐ and genetically induced obese mice. Similar to immune infiltration driven by disruption of endothelial junctions in response to pathogen exposure, neutrophils that underwent NETosis released amounts of ROS, which disrupts endothelial junctions and facilitates tumor extravasation. Mechanistically, ROS, along with MPO‐derived oxidant hypochlorous acid (MPO‐HOCl) dictates significant transcriptional reprogramming in the host ECs. In particular, the tight junction protein, junctional adhesion molecule‐1 was downregulated, which increased vascular permeability subsequently. Consistently, NETs inhibitors or genetic deletion of nitric oxide synthase‐2 reversed this effect in preclinical models of obesity. Together, these data mechanistically extend observations that activated innate immune cells modulate vascular integrity in the context of inflammation for metastatic extravasation.

#### 3.2.3 Awaken dormant cancer cells

Three distinct states of disseminated tumor cells in a metastatic organ have been described: proliferation, dormancy, and cell death. For some malignancies, metastatic extravasation at a secondary site occurs very early, whereas metastatic relapse takes several years or even decades. Increasing evidence suggests that these disseminated tumor cells undergo an extended period of dormancy in the stroma of target organs, where they resist therapy, escape immune surveillance, and survive in a quiescent state.^143^, ^144^ Ultimately, through mechanisms that are not yet understood, some of these cells are reawakened and become proliferative, forming lethal metastasis.^145^, ^146^ Despite strong clinical evidence linking dormant cancer cells with metastatic relapse, it is not known how to improve cancer outcomes in patients as these long‐lasting preserved cells are clinically undetectable, and mechanistic insight is lacking. A causal relationship has been demonstrated between systemic or pulmonary inflammation and the reawakening of metastatic dormant cancer cells, which is driven by alterations in the myeloid cell landscape and remodeling of ECM. A most recent study in a murine model bearing dormant breast cancer cells suggested that experimental pulmonary inflammation induced by repeated intranasal LPS instillation or tobacco smoke exposure triggered the reentry of dormant cancer cells to the cell cycle in a neutrophil‐dependent manner.^61^ NETs formed during inflammation engage in extensive and dynamic crosstalk with ECM, as well as the dormant cancer cells. Mechanistically, NET‐associated proteases, NE and MMP9, degrade laminin‐111, a vital component of the basement membrane ECM. The resulted conformational changes in the ECM activate FAK/ERK/MLCK/YAP signaling and awaken dormant cancers. During the degradation of laminin‐111, NET‐DNA acts as a proteolytic scaffold. Corroborating the importance of NETs in cancer cell awakening from dormancy, both disruption of NET‐DNA and inhibition of NE/MMP9 activity prevented LPS‐ and smoking‐induced cancer recurrence. This study first addresses this knowledge gap that activated neutrophils during chronic inflammation initiate the remodeling of ECM, trigger the integrin signaling of dormant cancer and awaken dormant cancer. Besides LPS‐ and smoking‐induced chronic inflammation, obesity is also associated with the elevated infiltration of neutrophils within lung tissue and an increased risk of tumor metastasis. Further characterization of NETs/ECM pathways in different inflammation models is warranted to verify that targeting these pathways should be clinically administered to reduce the risk of metastatic relapse.

### 3.3 NETs and cancer‐associated venous thrombosis

Venous thromboembolism (VTE), including deep vein thrombosis and pulmonary embolism, is highly consequential for cancer patients and responsible for their high morbidity and mortality. Multiple triggers in the context of cancer are likely to contribute to the occurrence of VTE, namely the production of tissue factors and compression of blood vessels, which together lead to the onset of a hypercoagulable state.^147^ Recently, neutrophil activation and the resulting NETosis have been increasingly reported in cancer‐associated VTE. Elevated serum levels of citrullinated histones increase the risk of venous thrombotic events in cancer patients,^148^ and arterial cancerous microthrombi in postmortem samples also contain citrullinated histones.^149^ Indeed, in the MMTV‐PyMT breast cancer and RIP1‐Tag2 insulinoma cancer models, renal tissue becomes poorly perfused due to increased levels of circulating NETs, and digestion of NETs with DNase I improves tissue perfusion.^150^

Recently, the issue of how NETs induce thrombosis has been intensively studied. In general, histones present on NETs can induce platelet recruitment and aggregation by increasing the secretion of Von Willebrand factor from ECs.^151^ Histones also activate platelets by acting as ligands for TLR2 and TLR4 on platelets.^152^ Proteases present on NETs, such as NE and CG, can promote thrombus formation by proteolytically inactivating tissue factor pathway inhibitors, which are strong anticoagulants in platelets.^153^ The sticky DNA structure of NETs itself can act as a scaffold to hold erythrocytes and activated platelets together to form thrombi.^154^ Finally, IL‐1β can increase the amount of circulating G‐CSF, leading to accumulated NETs in the circulation and ultimately more thrombus formation as mentioned.^105^ Inhibition of the IL‐1β receptor reduces G‐CSF levels and prevents thrombus formation in a mouse model of deep vein thrombosis.^105^

### 3.4 NETs and therapeutic resistance in cancer

#### 3.4.1 NETs and radio‐resistance

Although radiation therapy is an essential modality in the treatment of more than 60% of cancer patients, the incidence of radioresistance remains high clinically.^155^, ^156^, ^157^ Recent evidence suggests that the formation of NETs has a pivotal role in inflammation‐driven radiotherapy resistance.^51^ Shinde‐Jadhav et al.^51^ reported that in the mice bladder cancer model, NETs deposition is detected in the tumor immune microenvironment of mice treated with radiotherapy. When the tumor is irradiated, the release of HMGB1 from damaged tumor cells may lead to NETs formation in a TLR4‐dependent manner. In addition, the likelihood of developing radioresistance was increased, demonstrating an association between local response to radiotherapy, neutrophil status, and radioresistance.^51^ Inhibition of the production or degradation of NETs by NE inhibitor (NEi) or DNase 1 resulted in improved radiosensitivity of tumors, highlighting the role of these agents for potential synergy with radiation therapy.^51^ The research elucidated that tumor irradiation induced the production of NETs, which in turn played a functional role in radiation therapy resistance.^51^ Importantly, researchers demonstrated therapeutic relevance by reporting the presence of abundant NETs in tumor tissues of patients with poor radio‐response, predicting an unfortunate prognosis.^51^

#### 3.4.2 NETs and chemoresistance

Although few studies have examined the clinical link between circulating NETs levels and chemotherapy, preliminary data in vitro and in vivo support NETosis as a consequence of chemotherapy but also as a mechanism of chemoresistance. It suggests that in breast cancer, NETosis is responsible for the release of cell‐free DNA following doxorubicin and epirubicin treatment.^158^ Dr Nefedova's group^159^ reported that neutrophils exhibited potent chemoprotective effects and played a role in promoting the survival of multiple myeloma cells in the presence of doxorubicin. Mechanistically, the neutrophil‐dependent chemoprotection appears to be driven by soluble factors produced by TANs in TME.^159^ After that, the same group expanded upon these findings and revealed that NETosis is a mechanism for this neutrophil‐dependent multiple myeloma chemoresistance.^160^ NETs were shown to be internalized by tumor cells and subsequently bound to and detoxified by various anthracyclines such as doxorubicin, which can be reversed by DNase I treatment.^160^

#### 3.4.3 NETs interfere with immune cytotoxicity

Although cancer cells “per se” have potent mechanisms to induce neutrophils to expel NETs in the absence of infection, little information is available about the role of NETs in the tumor immune landscape and tumor response to immunotherapy. Highlighting the close relationship between intratumoral neutrophils and poor prognosis in patients with solid cancer, it seems logical, therefore, that cancer cells switch the functional state of neutrophils towards immunosuppression that protects tumor cells from cytotoxic immune attack. Teijeira et al.^63^ recently showed that tumor‐derived CXCL chemokines, namely CXCL‐1, ‐2, ‐5, ‐6, and ‐8, were capable of inducing NETosis in both human neutrophils and granulocytic MDSCs. Based on the advancement of intravital microscopy, TME imaging clearly showed the formed NETs shroud tumor cells and thereby impair contact of cytotoxic immune cells with tumor cells in vivo of a mouse model. To ascertain if the physical shield between tumor cells and cytotoxic effector lymphocytes impairs antitumor immune responses, they cocultured tumor spheroids with NETs and NK cells or CD8^+^ T cells. The number of surviving tumor cells was higher in the presence of NETs, suggesting that NETs protect tumor cells against cytotoxicity. Consistently, this protective mechanism was lost when extracellular DNA was digested by DNaseI. Furthermore, pharmacological inhibition of NETosis by intraperitoneal GSK484 administration sensitized tumors to PD‐1 and CTLA‐4 dual checkpoint blockade. Intriguingly, the induction of NETosis by tumor‐derived chemokines depends on CXCR1/CXCR2, which regulates leukocyte trafficking toward inflammation and transduces the Gi signaling. Pharmacological inhibition of CXCR1/CXCR2 significantly reduced NETs extrusion. Consistent with this finding, interventions to inhibit CXCR1/CXCR2 profoundly augment immunotherapy and suppress metastasis by depletion of intratumoral neutrophils/MDSCs in tumor‐bearing mice. Indeed, these findings provide the first definitive evidence that antitumor immunity could be improved by disruption of NETosis induced by tumor‐derived chemokines.

## 4 POTENTIAL THERAPEUTIC INTERVENTIONS

Based on current research that neutrophil depletion has its restrictions on the clinical application as a risk of severe infection, targeting NETs may be a potential strategy against tumors, given their pivotal role in inflammation‐driven cancer initiation and progression. The primary treatment approaches for blocking NETs include the prevention of NETs formation, destruction of NETs structure, or elimination of the interaction between cancer cells and NETs (Table 1).

**TABLE 1:** Inhibitors targeting NETs inducers.

Targets	Inhibitors	Treatment	Cancer
G‐CSF	Anti‐G‐CSF antibodies MAB414	Intraperitoneal injection	Breast cancer^150^
CXCR1/CXCR2	Reparixin /CXCR1 blocking antibodies	Intraperitoneal injection	Breast cancer^63^
	SX‐682	Orally	Metastatic melanoma (NCT03161431); metastatic colorectal (NCT04599140); metastatic pancreatic ductal adenocarcinoma (NCT04477343); advanced solid tumors (NCT04574583)
	Navarixin	Orally	NSCLC, castration‐resistant prostate cancer (CRPC), microsatellite stable (MSS) colorectal cancer (CRC) (NCT03473925)
	AZD5069	Orally	Metastatic castration‐resistant prostate cancer(mCRPC) (NCT03177187)
CTSC	AZD7986	Orally	Breast cancer^80^
PAD4	GSK484	Intraperitoneal injection/intravenous injection	Breast cancer^61^, ^80^, ^105^, ^161^; prostate cancer^61^; melanoma^86^; pancreatic cancer^86^; ovarian cancer^67^
	Cl‐amidine	Intraperitoneal injection	Breast cancer^60^, ^98^; melanoma^86^; pancreatic cancer^86^; HNSCC^98^
	BB‐Cl‐amidine	Intraperitoneal injection	Colorectal cancer^84^
	BMS‐P5	Orally	Multiple myeloma^162^
	YW4‐03	Intraperitoneal injection	Colorectal cancer^79^
Autophagy	Chloroquine	Orally	Pancreatic cancer^163^
	Hydroxychloroquine	Orally	Pancreatic cancer^163^
IL‐1β	IL‐1β‐neutralizing antibody	Intraperitoneal injection	Breast cancer^80^
	Selective inhibitor of IL‐1R, Anakinra	Subcutaneously	Breast cancer^105^
NEMO	NBD peptide	Subcutaneously	Breast cancer^164^
Enpp1	CM‐3163	–	Breast cancer^77^
Plasma redox balance	Albumin	Intravenous injection	HNSCC^98^; breast cancer ^98^
Anakinra	IL‐1R	Subcutaneously	Breast cancer subcutaneously^105^
Hypercoagulation	Enoxaparin	Subcutaneous injection in mini osmotic pumps	Small intestinal tumor^104^
Hypercoagulation	Warfarin	Orally	Small intestinal tumor^104^
Amyloid β	BACE inhibitor	Intraperitoneal injection	Skin cancer^86^
IL‐17	IL‐17 antibody	Intraperitoneal injection	Pancreatic cancer^88^
TLR4/9	Hydroxychloroquine	Orally	Hepatocellular carcinoma^165^
Inflammation	Aspirin	Orally	Hepatocellular carcinoma^165^
Inflammation	Lidocaine	Intravenous injection	Breast cancer^166^
DNA	DNase I	Intraperitoneal injection/intravenous injection/intramuscular injection	Breast cancer^60^, ^61^, ^80^, ^98^, ^150^, ^161^; HNSCC^98^; lung cancer^90^, ^91^, ^167^; colorectal cancer^79^, ^167^; pancreatic cancer^87^; prostate cancer^61^; hepatocellular carcinoma^165^; diffuse large B‐cell lymphoma^168^; ovarian cancer^67^
NE	Sivelestat	Orally/intraperitoneal injection	Lung cancer^167^; colon cancer^167^; breast cancer^61^, ^80^; prostate cancer^61^
	GW311616A	Orally	Lung cancer^90^; diffuse large B‐cell lymphoma^168^
CCDC25	CCDC25 antibody	Intravenous injection	Breast cancer^136^

Abbreviations: BACE, β‐Site amyloid precursor protein cleaving enzyme; HNSCC, head and neck squamous cell carcinoma; IL‐1R, IL‐1 receptor; NBD, NEMO‐binding domain; NEMO, NF‐κB essential modifier.

### 4.1 Blocking NETs formation

#### 4.1.1 Anti‐G‐CSF antibody

Evidence continues to accumulate suggesting that elevated levels of G‐CSF in tumor tissues or circulation correspond with NETosis and cancer‐related thrombosis in animal models, as well as in patients with cancer. The formation of intravascular NETs is responsible for poor peripheral vessel function in cancer patients. MAB414, an anti‐G‐CSF antibody, was supposed to break down the platelet–neutrophil complex of the peripheral vessel caused by tumors and improve vascular perfusion in the renal vasculature in the MMTV‐PyMT mouse model.^150^

#### 4.1.2 CXCR1/CXCR2 antagonists

Considering the predominant role of IL‐8–CXCR1/CXCR2 in NETosis, various clinical trials are presently underway intending to determine the efficacy of CXCR1/CXCR2 inhibitors in blocking NETs and controlling metastatic disease treatments. A selective inhibitor of CXCR1/CXCR2, Reparixin, drastically reduced the amount of NETs extrusion in 4T1‐bearing mice.^63^ An orally bioavailable inhibitor of CXCR1/CXCR2, SX‐682, blocks neutrophils recruitment and stimulates T cell anticancer response. In combination with PD‐1/CTLA‐4 dual ICIs Pembrolizumab and Nivolumab in several phases I/II clinical trials, the anticancer effect of SX‐682 is being assessed in metastatic tumors, such as melanoma (NCT03161431), colorectal carcinoma (NCT04599140), and PDAC (NCT04477343). In advanced solid tumors (NCT04574583), SX‐682 is evaluated to see if it has the potential to overcome resistance to antiprogrammed cell death ligand 1 (PD‐L1) agents M7824 when combined with the cancer vaccine CV301 TRICOM. Navarixin, one of the potent and selective antagonists of the human CXCR1/CXCR2, is also evaluated in combination with Pembrolizumab in NSCLC participants, castration‐resistant prostate cancer, or microsatellite stable colorectal cancer (NCT03473925). AZD5069 is a novel antagonist of CXCR2, which is shown to inhibit the binding of IL‐8 to CXCR2 specifically and is being investigated for the clinical benefits in combination with Enzalutamide in metastatic castration‐resistant prostate cancer patients (NCT03177187).

#### 4.1.3 CTSC inhibitor

Cathepsin C (CTSC), a cysteine protease that exists in lysosomes, is required for the catalytic capability of proteinase 3 and mediates NETosis via regulating the IL‐1b–p38–ROS axis of neutrophils.^80^ A second‐generation inhibitor of tumor‐derived CTSC, AZD7986, has been developed as a therapy for inflammatory lung diseases in clinical trials (NCT03218917). The mechanism by which AZD7986 suppresses NETs may also depend on the reduction of endogenous CTSC of neutrophils, which requires further exploration in metastasis treatment.

#### 4.1.4 PAD4 inhibitor

The discovery of a relationship between cancer and NETosis supports the rationale for targeting PAD4 as a treatment for patients with cancer. CI‐amidine,^60^, ^84^, ^86^, ^98^, ^164^ an irreversible pan‐PAD inhibitor, has efficacy against isozymes of the PAD family. GSK‐484,^61^, ^67^, ^80^, ^86^, ^161^ a reversible selective PAD4 inhibitor, demonstrates a similar effect to DNase I in blocking NETs formation and tumor progression. A pan‐PAD2/PAD4 inhibitor, YW4‐03, was also tested in a preclinical liver metastasis model after surgical stress. NETs‐induced inflammatory storms triggered by surgical stress are alleviated by YW4‐03.^79^ Nefedova et al.^162^ tried a novel PAD4‐specific inhibitor BMS‐P5 in myeloma‐bearing mice and found a more substantial effect on the inhibition of NETosis compared with other nonselective PAD inhibitors CI‐amidine and GSK‐484.

#### 4.1.5 Targeting autophagy

Additionally, NETs creation may also partially rely on autophagy, a process in which the cell eliminates superfluous or malfunctioning components via a lysosome‐dependent mechanism. As for targeting autophagy, chloroquine caused a reduction in NETs formation, a lower level of serum DNA, downregulation of citrullinated histone H3 expression, platelet aggregation, and tissue factor generation in the PDAC model.^169^ A phase I/II clinical study was conducted on patients who had neoadjuvant gemcitabine plus hydroxychloroquine treatment. Patients with decreased carbohydrate antigen 19‐9 (CA199) levels were observed a significant drop in cell‐free DNA in serum and lower amounts of citrullinated histone H3 in resection tissues. As for the significance of RAGE‐mediated autophagy, neutrophils genetically from RAGE‐deficient mice showed less proclivity to produce NETs than those from wild‐type mice.^163^

#### 4.1.6 Other blockers of NETs

As mentioned above, targeting an essential downstream molecule of CTSC, the IL‐1β antibody provides a similar effect as the CTSC inhibitors on preventing circulatory and pulmonary NETosis.^170^ To deeply explore the mechanism of NETs‐mediated breast cancer progression, NF‐κB signaling is an important NETs downstream pathway, by which NETs enhance the interaction between NEMO and IKK. Based on the mechanism above, Zhu et al.^164^ found NBD peptide, a selective inhibitor of NF‐κB blocked the formation of NETs in breast cancer xenograft and MMTV‐PyMT mouse models. Moreover, CM‐3163, a pharmacological Enpp1 blocking agent, can eliminate locoregional recurrence when combined with adjuvant irradiation in breast cancer patients postoperatively.^77^

Considering that ROS accumulation in neutrophils is a crucial step in the progression of NETosis, antioxidants may serve as preventatives for this process. Bratman et al.^98^ discovered that the endogenous antioxidant albumin was responsible for the redox state of plasma and abolished the NETosis and colonization of CTCs inside the lungs. A selective inhibitor of IL‐1R, Anakinra, is also shown to attenuate cancer‐associated thrombosis in mice bearing metastatic 4T1 tumors.^105^ As mentioned above, C3aR overexpression on neutrophils is involved in hypercoagulation and NETs formation. Research showed that anticoagulant therapies such as C3aR signaling blockage or LMWH administration negatively correlated with NETosis, leading to decreased tumor growth or enhanced tumor regression in mouse models.^104^

BACE inhibitor that targets β‐secretases, a protein with significance in both inflammatory disorders and the formation of tumor‐associated NETs, could potentially be leveraged to improve cancer treatment in skin tumor‐bearing mice.^86^ As previously stated, IL‐17 is a potent inducer of NETosis in pancreatic cancer due to immunomodulatory properties. Blocking IL‐17A and anti‐PD‐L1 treatment synergistically prevents NETosis and promotes antitumor immunity in mice.^88^

Several conventional anti‐inflammatory drugs have demonstrated effectiveness against solid tumors by hindering NETs in the inflammatory‐activated TME. Aspirin and hydroxychloroquine can reduce the NET‐triggered intrahepatic or lung metastatic capability of HCC by targeting cyclooxygenase‐2 or inhibiting upstream TLRs, particularly in combination with DNase I.^165^

A recent clinical trial was conducted to see whether the anesthetic or analgesic approach employed during tumorectomy affected the development of NETosis, subsequent tumor recurrence and survival outcomes. Anesthetic lidocaine, which is utilized for its analgesic and anti‐inflammatory qualities, has recently been demonstrated to prevent breast cancer recurrence by inhibiting postoperative expression of MPO and H3Cit in circulation (NCT02839668).^166^

### 4.2 Degradation of NETs structure

#### 4.2.1 DNase I

DNase I, the therapy for cystic fibrosis authorized by the United States Food and Drug Administration, has shown therapeutic value in preclinical models of breast cancer,^60^, ^61^, ^80^, ^98^, ^150^, ^161^ lung cancer,^90^, ^91^, ^167^ colorectal cancer,^79^, ^167^ pancreatic cancer,^87^ head and neck squamous cell carcinomas,^98^ hepatocellular carcinoma,^165^ diffuse large B‐cell lymphoma,^168^ prostate cancer,^61^ as well as associated metastatic niches through destroying NETs. Encouragingly, several prospective clinical trials are currently evaluating the benefits of DNase I administration in the treatment of cancer. Pulmozume, a recombinant human DNase, is now being evaluated for its safety, tolerability, and therapeutic efficacy in a randomized, placebo‐controlled trial in a population with stage III–IV head and neck cancer (NCT00536952). In another phase II study, patients with acute myeloid leukemia or lymphoid leukemia were administrated with a combination of Oshadi D (DNase in an Oshadi carrier) and Oshadi R (RNase in an Oshadi carrier) for salvage chemotherapy, which exhibits potential clinical application value (NCT02462265).

#### 4.2.2 NE inhibitor

Given the extensive role of NE in the initiation of chromatin decondensation, pharmacologic inhibition of NE by Sivelestat^61^, ^80^, ^167^ in preclinical lung and gastrointestinal tumor models prevented liver and lung metastasis. GW311616A is orally accessible with a long half‐life and high affinity that interferes with the activity of NE.^71^, ^90^ Systemic administration of GW311616A following cecal ligation and puncture abrogated hepatic metastasis by NETs prevention. As compared with GW311616A, both DNase I and PAD4 inhibitors face the challenge of a short half‐life in circulation, which limits their therapeutic utility of systemic administration.

### 4.3 Interfering with the interaction of cancer cells with NETs

It has been proposed that the cancer cell transmembrane protein CCDC25 acts as an extracellular DNA receptor, which detects NET‐DNA structure and attracts breast cancer cells to form liver metastasis. Therefore, the CCDC25 antibody will be an effective therapeutic strategy to interrupt the formation of the CCDC25–NET complex and metastasis in the mouse model.^136^ The expression of integrin on both tumor cells and neutrophils is regarded as a crucial modulator in promoting tumor–NET interaction. Adoptive transfer of neutrophils deficient in IL‐β1 resulted in a substantial reduction in hepatic sinusoid adhesion of lung cancer cells.^91^ By mass spectrometric analysis of purified NETs, several candidate proteins were discovered involved in the capture and dissemination of cancer cells, as well as the promotion of NET‐facilitated metastatic spread. Carcinoembryonic antigen cell adhesion molecule 1 (CEACAM1), a member of the carcinoembryonic antigen (CEA) family, was discovered to increase the adherence and migration of metastatic colon cancer cells in a recent study. Consistently, anti‐CEACAM1 therapy is a promising treatment to abort these prometastatic connections between NETs and cancer cells.^171^

### 4.4 Combining NET‐blocking therapy with established anticancer treatments

Chemotherapy, radiotherapy, and immunotherapy constitute the crucial cancer‐treatment modalities. However, very few therapeutic alternatives are available for patients who do not respond to their first‐line treatments, which poses a significant obstacle in the fight against cancer. There is a substantial correlation between neutrophil infiltration within TME and the aggressiveness of the disease as well as resistance to treatments. Recent studies suggest that NETs‐blocking agents have the potential to improve the efficacy of conventional therapeutics including surgery, radiotherapy, and chemotherapy. In light of the significance of NETs in chemo‐, radio‐, and immunotherapy, it is vital to explore multiple combinations involving NETs in future clinical trials. Apart from that, NETs also perform an immunosuppressive role as a cause of ICI resistance. IL‐17‐induced NETosis shielded tumor cells from T‐cell immune attack. As a result, treating patients with pancreatic adenocarcinoma with IL‐17 blockade may help circumvent ICI resistance. Similar immunotherapy‐resistant effects were also demonstrated by Teijeira et al. based on their findings of activation of NETs via ligands of CXCR1/CXCR2^63^. As stated previously, CXCR1/CXCR2 antagonists have been conducted in combination with ICIs to reinvigorate immune attacks. Collectively, these findings support that NETosis interventions could potentially be leveraged to overcome immunosuppression and resistance to conventional therapies for cancer, but further investigation of their combinational therapeutic value is warranted.

## 5 CLINICAL UTILITY OF NETs AS A CANCER BIOMARKER

NET‐associated molecules can be detected in the circulation or tumor tissue, which have been increasingly regarded as diagnostic and prognostic biomarkers (Table 2). For patients with advanced gastric cancer, the presence of NETs in the peripheral blood, as a negative independent predictor of progression‐free survival, has a superior diagnostic ability to CEA and CA199.^172^ Additionally, circulating NETs negatively correlate with treatment response in advanced‐stage cancer patients receiving first‐line therapy. PD‐1 inhibitors have become a useful strategy for NSCLC patients; however, only a subset of these patients respond well. According to Huang et al.^173^ investigation into biomarkers that potentially predict the efficacy of PD‐1 inhibitors, patients with greater serum NET levels have worse outcomes. NET‐related genes in TCGA have been identified as predictors of overall survival, immunotherapy efficacy and are also associated with an immunosuppression microenvironment.^174^, ^175^ Additionally, NETs signature also could identify the population of tumor patients most likely to respond favorably to treatments such as neoadjuvant therapy^176^ or Wnt signaling pathway inhibitors.^177^ Others attempt to establish a robust NETs‐related long noncoding RNA (NETsLnc) signature for predicting the prognosis of soft tissue sarcoma by integrating machine learning algorithms.^178^ Likewise, NET‐related molecules have the potential to serve as biomarkers of activated platelets, which promote VTE and accelerate tumor‐associated thrombosis.^64^, ^104^, ^179^ Clinical trials have been established to investigate the potential of NETs as a biomarker for the prediction of VTE in patients with tumors. The formation of NETs associated with treatments has been investigated in clinical research; however, the majority of these studies are observational and require prospective studies. Consequently, accurate measurement of NETs provides diagnostic, therapeutic, and prognostic information for malignancies (Table 3).

**TABLE 2:** NET‐related molecules serve as tumor biomarkers.

Marker	Site	Cancer
MPO–DNA	Circulation	Colorectal cancer^78^, ^79^, ^180^; liver cancer^180^; gallbladder cancer^51^; lung cancer^167^, ^181^; esophagogastric cancer^167^; gastric cancer^182^; breast cancer^136^; pancreatic cancer^183^; multiple advanced cancer^184^
cfDNA/cfmtDNA	Circulation	Endometrial cancer^185^, ^186^; gastric cancer^182^; pancreatic cancer^179^, ^183^, ^187^; multiple advanced cancer^184^, ^188^; colorectal cancer^189^; breast cancer^64^, ^190^
NE–DNA	Circulation	Breast cancer^191^; gastric cancer^172^
Putative DNA	Circulation	Breast cancer^64^
Nucleosomes	Circulation	Gastric cancer^182^; multiple types of cancer,^188^ pancreatic cancer^179^
NE	Circulation	Multiple advanced cancer^184^; gastric cancer^182^; pancreatic cancer^187^
H3Cit	Circulation	Multiple advanced cancer^184^, ^188^; pancreatic cancer^187^; breast cancer^64^
MPO	Circulation	Pancreatic ductal adenocarcinoma and distal extrahepatic cholangiocarcinoma^179^; multiple advanced cancers^184^
HMGB1	Circulation	Colorectal cancer^79^
Calprotectin	Circulation	Pancreatic ductal adenocarcinoma and distal extrahepatic cholangiocarcinoma^179^
G‐CSF	Circulation	Head and neck cancer^66^; pancreatic cancer^187^
H3Cit	Liver ischemic lobes	Colorectal cancer^79^
H3Cit	Metastatic lesions	Breast cancer^136^; colorectal cancer liver metastasis^180^
H3Cit	Primary tumor	Breast cancer^63^, ^136^; pancreatic cancer^44^, ^183^; Lewis lung carcinoma^65^; liver cancer^180^; colon cancer,^192^ gastric cancer^172^
CD15	Primary tumor	Pancreatic ductal adenocarcinoma^44^
MPO	Metastatic lesions	Breast cancer^136^
MPO	Primary tumor	Breast cancer^136^
NE	Primary tumor	Gastric cancer^172^

Abbreviations: cfmtDNA, cell‐free mitochondrial DNA; CD15, cluster of differentiation 15.

**TABLE 3:** List of clinical trials with NETs on tumors.

Tumors	Functions	ClinicalTrials.gov ID
Hepatocellular Carcinoma	Predict portal vein tumor thrombosis	NCT05040347
Breast cancer	Mechanism of NETs formation induced by long‐term tamoxifen (TAM) treatment	NCT05056857
Pediatric solid and hematological malignancies	NETs formation following chemotherapy	NCT01533779
Solid cancer	NETs associated with venous thromboembolic events	NCT04294589
Occult cancer	Predict venous thromboembolism	NCT03781531

*Data sources*—ClinicalTrials.gov.

### 5.1 NETs biomarkers in circulation

The quantity of cfDNA has been utilized to determine the number of NETs released into the bloodstream. As a noninvasive, cost‐effective surrogate, it provides supportive information on diagnosis, prognosis, and disease progression for colorectal cancer,^189^ breast cancer,^64^, ^190^ gastroesophageal cancer,^182^ pancreatic cancer,^179^, ^183^, ^187^ and endometrial cancer.^185^, ^186^ Even though circulating cfDNA is associated with tumor burdens, it does not serve as a reliable NETs indicator. In addition to cancer cells, a number of additional variables, including apoptotic and necrotic cells caused by other diseases, could also contribute to elevated cfDNA levels in the plasma. The nucleosome consists of histones and double‐stranded DNA, a type of cfDNA. Several studies demonstrated that a lower nucleosome level prior to the second and third cycles of chemotherapy was associated with a favorable response to treatment in NSCLC patients.^193^ Both NE^66^, ^184^, ^187^ and MPO,^179^, ^184^ two components of NETs, are produced upon neutrophil activation but not NETs formation, indicating that more specific markers need to be explored. Moreover, recent studies have reported that serum levels of H3Cit, as well as circulating MPO–DNA or NE–DNA complexes, are associated with the diagnosis and/or progression of multiple cancers with much more specificity.^51^, ^78^, ^136^, ^148^, ^167^, ^179^, ^180^, ^184^, ^188^, ^191^, ^194^ These markers differentiate NETs from other types of nontumor‐derived cfDNA, which increases the likelihood of therapeutic translation. MPO–DNA or H3Cit has been employed in clinical trials as NETs biomarkers and prognostic indicators to evaluate the risk of thrombosis in patients with cancer including myeloproliferative neoplasms, pancreatic, gastric,^182^ and colorectal cancer.^79^

### 5.2 NETs biomarkers in tumor tissues

Increased H3Cit, NE, and MPO, indicators of NETosis, have been demonstrated in both the primary tumor^44^, ^63^, ^65^, ^172^, ^183^ and metastatic lesions.^79^, ^180^ However, the assay to assess tumor‐associated NETs in tissues remains in its infancy since it cannot be quantified reliably. Nowadays, another attempt to discover the link between NETs and the clinical outcomes of patients with cancer was afforded. By the investigation of their transcriptomes, the Least Absolute Shrinkage and Selection Operator (LASSO) Cox regression model was then used to create a 19‐gene NETs score. They showed that transcriptome‐based NETs scores had a negative correlation with prognosis in patients with cancer.

## 6 CONCLUSIONS AND PERSPECTIVES

Increasing clinical and preclinical evidence has provided insights into the NETosis induced by the cancer cell itself or its surrounding environment. Reciprocally, the formation of NETs can further support tumorigenesis and metastasis. Subsequent mechanistic studies have revealed the intricate interactions between NETs, cancer, and immune cells in TME, which should eventually lead to more innovative anti‐NETosis cancer therapies reaching the clinic. Given our improved understanding of NETs connected to metabolic remodeling in immune cells and immunosuppression, therapies against NETosis can not only repress metastatic colonization, but also restore cancer immune surveillance. Nevertheless, many questions concerning anti‐NETosis cancer therapies need to be addressed in future studies.

First, NETs comprise a complex structure of de‐condensed DNA and proteins of nuclear, granular, and cytosolic origin. The proteins associated with NETs are essential for the execution of complex processes such as NE‐ and MMP9‐mediated degradation of laminin‐111, resulting in remodeling of ECM and dormant cancer cells awakening. Other functional proteins within NETs responsible for tumor progression, including primary growth and metastasis, have been identified. It should be noted that disruption of the DNA backbone of NETs with DNase I, rather than DNA‐complexed granular proteins, represents an attractive strategy for cancer prevention and treatment. It is likely that DNA and protein within NETs integrate as a whole and, accordingly, strengthen the multivalent interactions with other molecules (proteins or small‐molecule substrates). Interestingly, NET‐DNA has been proposed to act as a proteolysis scaffold. Moreover, DNA‐scaffolded granular proteins, such as MPO, NE, and calgranulin B, are less susceptible to proteolytic degradation than their free counterparts. Future research would need to take into account the nature of NETs structures and that no single component is solely responsible for their activity.

Second, the formation of NETs also plays a vital role in host immune defense by entrapping pathogens. Systemic inhibition of NETs is unfavorable for the intrinsic defense mechanisms and may lead to increased susceptibility to bacterial infections, which are an essential cause of death in cancer patients. There is great motivation for cross‐disciplinary research to develop a controlled drug delivery system, which offers both the potential for spatial and temporal control of drug release. Intelligent materials have been designed, allowing for the periodic and localized release of DNase I cargos in the mPDA shell, triggered by NIR‐II light irradiation.^195^ The on‐demand release of DNase I with precise spatiotemporal control can eliminate extracellular NET‐DNA in both primary colorectal cancer and metastatic tumors.

Third and finally, further clinical trials are needed to identify and validate cancers that are closely linked to NETosis. Despite significant progress in understanding the contribution of NETs to tumor progression, the feature is strictly tissue‐specific. The most commonly reported organs in the literature of NETs deposition are the liver, as well as the lung. The recruitment and activation of neutrophils usually take place in postcapillary venules of the systemic microcirculation. The discovery of NETs in primary tumors and metastasis will significantly increase our current understanding of the pathogenic and prognostic functions of NETs in vivo, which is currently restricted by technologies for rapid and sensitive detection. Subgroups of PMNs including antitumorigenic N1 neutrophils and protumorigenic N2 neutrophils may form functionally distinct NETs. Apart from neutrophils, mast cells, eosinophils, basophils, and macrophages present in TME can also release extracellular DNA traps in limited quantities. A better understanding of the discrimination across different cells and improved diagnostic procedures warrant further investigations while researching to identify and characterize NETs in primary tumors and metastasis. Moreover, it is postulated that NETs may exert an anticancer effect via direct cytotoxic effects against cancer cells or by inducing an immune response against the tumor.^196^, ^197^ MPO, an essential component, is capable of destroying melanoma cells.^198^ Another crucial component of NETs, histones, have the ability to harm ECs of the blood vessels that supply the tumor.^199^ Hence, researchers should pay more attention to the double‐edged of NETs in tumors.

In conclusion, clarifying the role of NETs in carcinogenesis and tumor progression will not only enhance our understanding of disease pathogenesis, but will also enable the identification of biomarkers that can discriminate patients with cancer at risk of progression to metastasis, venous thrombosis, and therapeutic resistance. New insights into the role of NETs also provide the rationale for novel antitumor therapies.

## AUTHOR CONTRIBUTIONS

Jinghua Ren had the idea for the article. Yuxi Ma and Jielin Wei performed the literature search and drafted the article. Jinghua Ren and Wenshan He critically revised the work. All authors have read and approved the final manuscript.

## CONFLICT OF INTEREST STATEMENT

The authors declare no conflict of interest.

## ETHICS STATEMENT

Not applicable.

## Data Availability

Data sharing is not applicable to this article as no datasets were generated or analyzed during the current study.
